# Evolution of type-II hetero-strain cylindrical-gate-all-around nanowire FET for exploration and analysis of enriched performances

**DOI:** 10.1038/s41598-023-38239-x

**Published:** 2023-07-14

**Authors:** Rasmita Barik, Rudra Sankar Dhar, Falah Awwad, Mousa I. Hussein

**Affiliations:** 1 Department of Electronics and Communication Engineering, National Institute of Technology Mizoram, Chaltlang, Aizawl, Mizoram India; 2 Department of Electrical Engineering, United Arab Emirates University, Al Ain, United Arab Emirates

**Keywords:** Engineering, Nanoscience and technology

## Abstract

The incubation of strained nano-system in the form of tri-layered structure as nanowire channel in the cylindrical-gate-all-around (CGAA) FET at 10 nm gate length is developed for the first time to keep abreast with the proposed 3 nm technology node of IRDS 2022. The system installs Type-II hetero-strain alignment in the channel attesting itself as the fastest operating device debasing the SCEs at nano regime. The ultra-thin strained-channel comprises of two cylindrical s-Si wells encompassing s-SiGe barrier in between, which enables improvement of carrier mobility by succumbing of quantum charge carriers in the region. This results in 2D charge centroid creation with cylindrical based circular Nano-system contemplating electrostatic potential difference leading to enriched electric field, current density and transconductance, while the gate-all-around architecture with increased gate controllability lowers leakage current, in the device. The 10 nm strained-channel CGAA astounded havoc ON current enhancements of ~ 20% over 22 nm strained CGAA, 57% over Si CGAA FET and 75% over proposed 3 nm technology node IRDS 2022 are accomplished. Hence, carrier mobility and velocity enriches instituting quasi-ballistic transport through the Nanowire channel, thereby augments in ~ 28% drain current so the 10 nm channel CGAA FET stands as the most suitable and improved device in nano regime.

## Introduction

Enhancing electron mobility, on-current, and transconductance by using strain technology in a nanodevice channel improves overall device performance^1^, but optimising short channel effects (SCEs) beyond the 14 nm technology node is a challenge. These SCEs affect power dissipation and provide significant variations to electrical characteristics rasing off-current leakages in nano regime^2^. Numerous approaches, including mobility enhanmcent using strain technologgy^3^, high-k dielectrics metal gate^4^, optimal doping profile design^5^, quasi-ballistic FETs^6^, and Nanowire (NW) FETs^7^, are researched in order to boost transistor performance and lower device variability. According to the literature, global strain and local strain generates biaxial and uniaxial strain, respectively^8^, bringing about designs such as: strain-silicon on relaxed SiGe^9^, dual-layer strained channel (s-Si/s-SiGe)^10^, Strain-silicon on insulator (SSOI)^11^, and Hetero-structure on insulator (HOI)^12^. In order to bridge the energy band gap for improved electron mobility, silicon is subjected to increased strain, which lowers the threshold voltage (V_th_)^10^. It is well-known that the strain strategies have no long-term reliability impact and barely affect the gate oxide quality^13^. The application of strain in nanoFETs results in mainly two effects: (i) shift in band energy and (ii) degeneracy splitting of the electronic states within the structure. The conduction band edge in the fourfold valley is higher in energy than the twofold valley due to strained channel^1^, instigating energy band splitting and increasing occupancy of electrons in the twofold valleys. This condition results into forming a twofold degenerate energy band, which in turn congregates in enhanced electron mobility, suppressing the intervalley transition of electrons from lower valley to upper valley reducing the phonon scattering in the ultrathin channel. In order to develop new hetero-structure devices, the concept of strain technology plays a crucial part in current FET technology, because it increases drain current without requiring any additional scaling, which is sufficient for further device performance improvements. Thus, these technology-based designs became more industrialised during the past years despite the fact that the issue of device controllability at the nano regime persists.

On the other hand, numerous gate devices that are electrically controlled by a single gate electrode have emerged, including DG FETs, FinFETs, and Gate All Around (GAA) FETs^14^. These multi-gate architectures developed to improve gate controllability and, to some extent, reduce leakage currents. One of the promising modern technologies is the gate all around (GAA) nanowire FET, which is a scaled-down MOSFET. Cylindrical GAA (CGAA) is a type of gate-all-around device that offers better functioning than rectangular GAA (RGAA) FET among the several gate-all-around devices. Due to electrostatic coupling between adjacent gates at the corners, which results in current crowding, the RGAA FET shows undesirable characteristics known as corner impact^15^. This problem is severe as the device drops below 14 nm in gate length due to the induction of quantum effects, and the device is thus susceptible to quasi-ballistic phenomena, hence not a prolific design. Although, Cylindrical GAA devices stand out and are regarded as superior^16^, issues like the narrow-channel effect^17^ causes lower mobility and electric field to minimize performance making it difficult to acquire the desired results. Hence, the researchers are constantly striving to reinvent the device with new technologies and material artefacts by developing improved CGAA in the nano domain.

Few technologies needs to be merged in order to increase the viability of FETs in terms of improved performance at nanoscale. As a result, one feasible option is to augment strain engineering by introducing the tri-layered strain Nano-system^18^ to combat the barrier potential down-pulling induced by the multiple SCEs in the technologically advanced CGAA FETs to meet up for IRDS 2022^19^ Research on development of dual-gate all around core–shell nanotube TFET^20^ explores the dominant features and characteristics of the dual-gate all around core–shell nanotube TFET architecture in contrast to the Nanowire architecture. The gate length and gate thickness of the device is ~ 50 nm and ~ 10 nm, respectively, hence no way a match for today’s technological arena. Now, with the device being scaled down to nano regime undesirable SCE accumulates. To overcome these SCEs and to be at par with the IRDS 2022, a novel strained channel cylindrical GAA FET structure is being proposed. This structure is expected to provide better gate control, superior performance as compared to DG FET, Fin FET, conventional GAA FET and core–shell GAA FET. The novel tri-layer strained channel Nanowire (NW) CGAA at the 10 nm and at 22 nm gate length are proposed and developed in this work. The 10 nm gate length device is settled to provide enriched performance in comparison to the proposed 3 nm technology node device of IRDS 2022^19^. The novel structure incubares a heterostructured system and is anticipated to deliver improved performance through faster switching as a result of enhanced electron mobility in the narrow-channel likely to lead to proposedly quasi-ballistic transport, carrier confinement, and reduced scattering effect, enhancing on-current outcomes while controlling leakage pathways. The device structure and design is based on the physics of material relics, which is further explored and a detailed analysis and investigation of the novel CGAA FET is established and presented here.

## Device structure and theory

**Figure 1:**
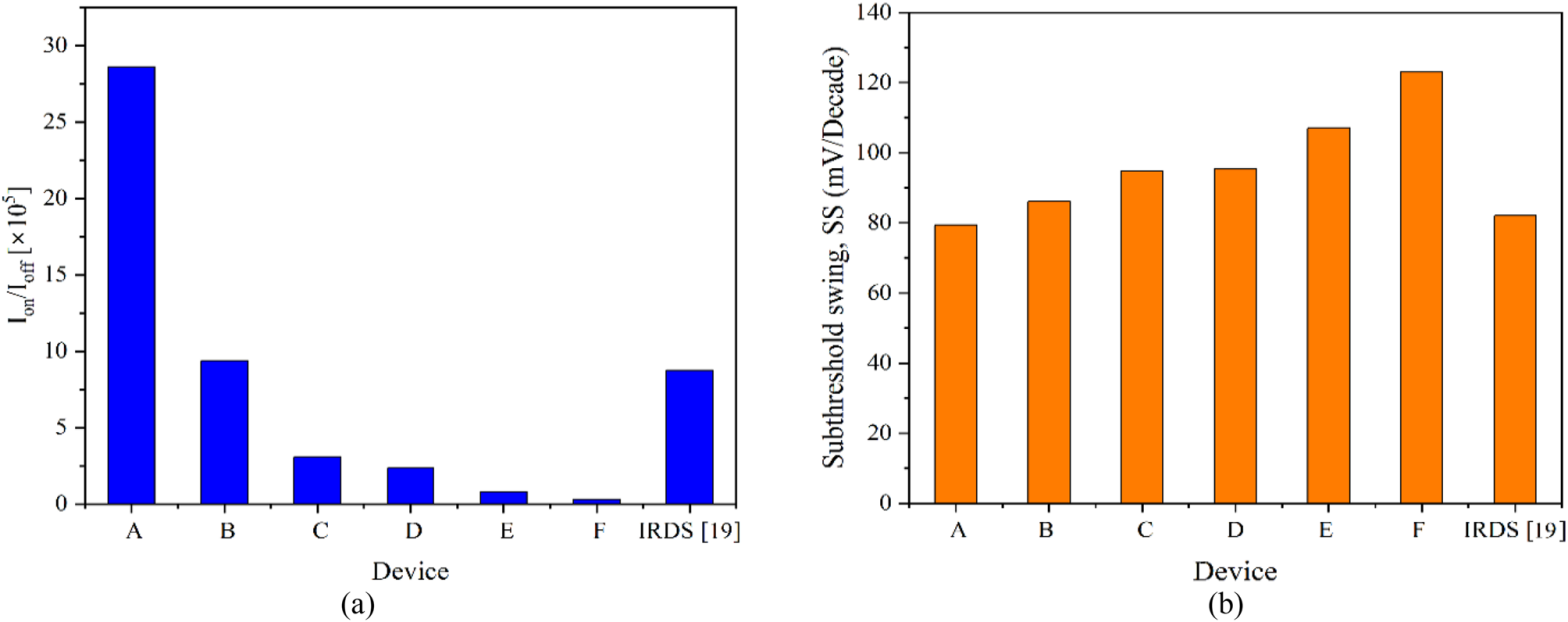
Device A to Device F (10 nm gate length) are compared with proposed 3 nm technology node of IRDS 2022 (**a**) I_on_/I_off_ current ratio. (**b**) Subthreshold swing.

The innovative 10 nm gate length CGAA based device is developed with variations considering the thickness of the internal channel layers (s-Si/s-SiGe/s-Si) and is labelled as Device A to Device F as listed in Table 1. Maintaining the s–Si layer thickness at 1 nm the s-SiGe thickness is varied as 2, 2.5 and 3 nm forming Device A, B, and C, respectively. Thereafter, keeping the s-Si layer fixed at 1.5 nm, Device D, E, and F are developed with s-SiGe thicknesses as 2, 2.5 and 3 nm, respectively. Reducing the s-SiGe thickness below 2 nm installs probable misfit dislocations in the atomic layer structure, hence avoided. The thickness of silicon layer of the newly developed devices is maintained in between 1 and 1.5 nm as was suggested feasible by Leys et al.^21^ which also have the scattering effect under control in the ultra-thin channel. Another Device G is developed with this novel technology-based nano-channel system for the 22 nm gate length having radial thickness of the channel layers as 1.5–2–1.5 nm. This device is mainly employed for comparison with the existing 22 nm Si only channel CGAA (Device H) of Karbalaei et al.^16^. Also, Device I (a Tri-gate HOI FinFET) of Nanda et al.^22^ is utilized having the tri-layered nano-channel system at 10 nm gate length for comparison. Device H and Device I are utilized for calibration, validation and investigation for performance enhancement of the present work. All these devices are tabulated and presented in Table 1. The I_on_/I_off_ current ratio, which mostly determines the device performance (the higher the better), and the subthreshold swing (SS), that defines the leakage quantity occurring in the device (a major SCE at nano regime), of Device A to Device F are analysed and observed and tabulated in Table 2 as also overlaid in Fig. 1a, b, respectively, which clearly indicates that Device A stands to be superior among all the 10 nm gate length devices, hence the other devices can be discarded. This is attributed to the fact that with thicker s-SiGe layer density of hole carrier accumulation increases which in turn recombines with the transporting electrons of the s-Si well-layers leading to higher leakage and reduction of ON current as also signified from Fig. 1a, b, therefore, Device B, C, E, and F can in no way suffice close to Device A. In case of Device D the s-Si layer being wider leads to the fact that the Si layers are not fully strained and thereby the mobility is reduced, which directly installs in performance degradation. It is to be noted in Fig. 1 the comparison is presented also with the proposed 3 nm technology node device of IRDS 2022^19^ and Device A showing 3.7% decrement in SS and an enormous enhancement 227% in I_on_/I_off_ ratio is observed to be far superior, indicating that the novel device is a device of future era. Hence, the tri-layered nanowire (NW) channel CGAA device with 10 nm gate length having the channel radial thickness as 1–2–1 nm for s-Si/s-SiGe/s-Si layer (Device A) is observed to be healthier and an enriched device among the others compared.

**Table 1:** Device specification features.

Device	Device specification	Radial thickness of channel (s-Si/s-SiGe/s-Si) (nm)
A	10 nm tri-layer strained channel CGAA FET	1–2–1
B	10 nm tri-layer strained channel CGAA FET	1–2.5–1
C	10 nm tri-layer strained channel CGAA FET	1–3–1
D	10 nm tri-layer strained channel CGAA FET	1.5–2–1.5
E	10 nm tri-layer strained channel CGAA FET	1.5–2.5–1.5
F	10 nm tri-layer strained channel CGAA FET	1.5–3–1.5
G	22 nm tri-layer strained channel CGAA FET	1.5–2–1.5
H^16^	22 nm Silicon channel CGAA FET	6.4
I^22^	10 nm Tri-gate FinFET	1.5–3–1.5

**Table 2:** Comparision of varied NW channel CGAA FETs.

Device	I_on_/I_off_ (× 10^4^)	Subthreshold swing (mV/decade)
A	28.61	79
B	9.35	86
C	3.07	95
D	2.32	95
E	0.78	95
F	0.28	123
IRDS 2022^19^	8.74	82

The n-channel GAAFET with physical gate length of 10 nm is designed in three steps namely meshing, material filling and doping. The structure is segmented into various regions depending upon the material to be filled in the particular sections of generated meshes. The silicon is used as dopant, silicon oxide as gate dielectric and polysilicon is used as gate contact in the device. The channel region is designed with epitaxial growth forming the three-layer strained Nanosystem. The inner and outer layers are Silicon while the middle layer of Si_1−x_Ge_x_ is utilized having a mole fraction of x = 0.4 grown in between. So that the two sides as well as the upper layer of the central SiGe region are covered with Si to fabricate the strained hetero-channel regions. The two n-doped silicon regions are then grown on either sides of the channel region which forms the source and drain of the device, therefore developing the strained channel CGAA FET.

**Figure 2:**
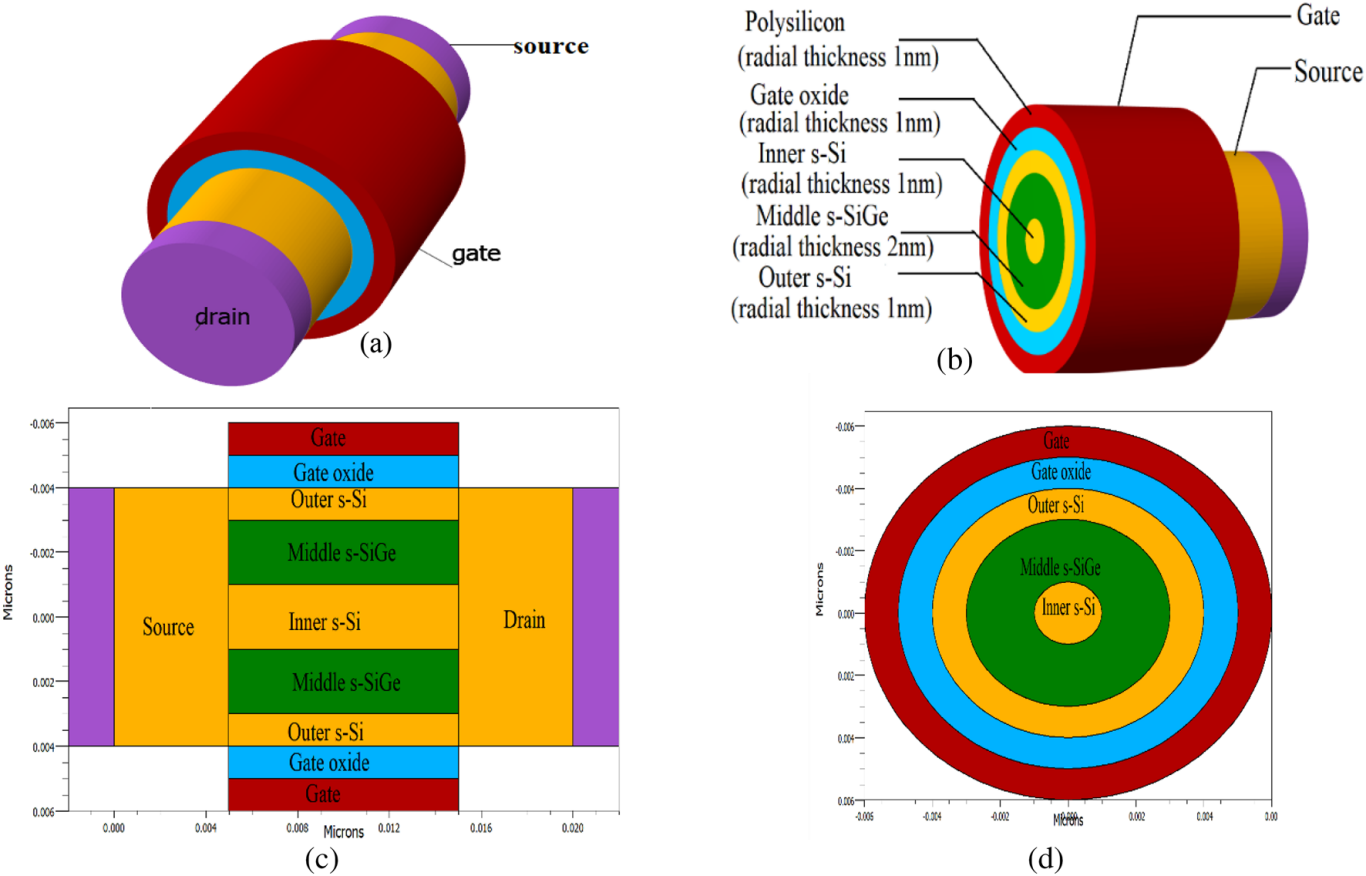
(**a**) Schematic 3D view of strained tri-layer channel cylindrical GAA FET. (**b**) The cut section view of the tri-layered strained channel in the device. (**c**) Top view of the CGAA FET. (**d**) 2D circular cross-sectional view of the tri-layered strained channel CGAA FET.

A 3D schematic of the narrow channel cylindrical GAA FET is created here on the 10 nm gate length (Device A) as in Fig. 2a. Two cylindrical well-based layers, one serving as the outer layer and the other as the inner layer, each 1 nm thick, are used to create the strained tri-layered channel in cylindrical GAA. These layers, which are defined as outer s-Si, and inner s-Si in Fig. 2b, are tensile strained as a result of the influx of the compression strained in the middle SiGe barrier layer because of Type-II heterogeneity, though the inner s-Si layer acts as a virtual substrate for the device and is merely strained due to the fact that there is no other substrate for the device development. The strained Si_x_Ge_1−x_ layer with x = 0.4 mol fraction serves as a 2 nm thick barrier in the nano-channel between the two s-Si layers. Figure 2c shows the top view of the entire device in cut plane condition, while a circular cross-sectional view of the channel is shown in Fig. 2d. Because the holes are not entirely contained at the surface the increased Ge concentration has a lessened effect due to the ultrathin s-Si surface layer. This strain engineering is prepared in an effort to perchance improved carrier mobility^23^, which will endure healthier performance than the contemporary silicon-only CGAA FETs. Additionally, the two strained Si layers that provide the encompassed wells for carrier confinement occurring in the SiGe layer across the cylindrical system are fitted in the device to further enhance electron transport in the device. Improving carrier mobility, transconductance, and electric field, counter threshold voltage roll-off occurs by consolidating the channel with these strained layers that indulges in carrier confinement and quasi-ballistic transport.

The 10 nm gate length NW CGAA dimensions and operational parameters considered here and enumerated in Table 3. The geometric specifications of the strained tri-layer (Si/SiGe/Si) dictate the radius as 1–2–1 nm in the channel for Device A, while the length is maintained at 10 nm, keeping the 2:1 length to thickness ratio so as to stabilize the threshold voltage and reduce SCEs. To control DIBL in the device within limits, the source and drain regions are also doped differently and the device is manifested to be enriched with respect to the proposed 3 nm technology device of IRDS 2022^19^. The tri-layered channel that forms the Nano-system structure of the device (except Device H) causes strain and band bending in the channel area. For s-Si, the increase in bandgap is governed by:

1 $${E}_{g}=\frac{{\eta }^{2}{\pi }^{2}}{2{m}_{r}{t}_{s{\text -}Si}^{2}}$$

where η = ℎ(2π)^−1^, ℎ = 6.6 × 10^–34^ J s, t_s-Si_ is the strained silicon layer thickness and ${m}_{r}=\frac{1}{{m}_{e}}+\frac{1}{{m}_{h}}$ where m_e_ is the electron effective mass and m_h_ is the hole effective mass. Karthik et al.^24^ analyzed the band structure of Si_1−x_Ge_x_ based on 4.2% lattice mismatch with Si, which refers to the change in charge density in valence and conduction band and is expressed as a function of mole fraction (x) given below:

2a $${\mathcal{\rm P}}_{siGe}\left(x\right)=11.8+4.2x$$

2b $$\Delta {{\rho }_{g}}_{s{\text -}SiGe }(x)=0.467 x$$

2c $$\Delta {{\rho }_{v}}_{s{\text -}Si }(x)=0.74 x$$

2d $$\Delta {{\rho }_{c}}_{s{\text -}SiGe}\left(x\right)\approx 0 x$$

where (ρ_g_)_s-SiGe_ indicates the natural reduction in the bandgap energy of SiGe from silicon and (ρ_c_)_s-SiGe_ denotes the shift in SiGe conduction band as a result of strain, and where ${\mathcal{\rm P}}$_SiGe_ is the permittivity of SiGe. Based on this concept, Kumar et. al.^25^ calculated the strain engineering effect for a DG FET nano-system, which is implemented here and the total strain in the tri-layered strained CGAA FET is calculated to be:

3 $${\Psi }_{ch{\text -}strain}=\sum \pi .{\it{f}}({R}_{eff}{)}^{2}$$

4 $${\mathrm{where } \quad R}_{eff}=\frac{{r}_{up s-Si}-{r}_{m SiGe}+{r}_{Ins{\text -}Si}}{{r}_{up s{\text -}Si}-{r}_{SiGe}}$$

and r_up s-Si_ is the radius of outer strained silicon, r_m SiGe_ is the radius of middle strained SiGe and r_in s-Si_ is the radius of inner strained silicon while Ψ_ch-stain_ is the total biaxial strain in the Nano-channel system developed here as a function of R_eff_ and is thus the effective radius based on radial thicknesses in the Nanowire channel that embraces the three ultra-thin layers (outer strained Silicon, middle strained SiGe and inner strained Silicon), forming a 2D circular tri-layered channel as illustrated in Fig. 2c. Ψ_ch-strain_ is the summation of total strain among the wells formed in 2D throughout the length of the channel of the device creating the cylindrical GAA FET.

**Table 3:** Parameter specifications of device a.

Description	Symbols	Values
Strained silicon radial thickness	t_s-Si_	1 nm
Strained SiGe radial thickness	t_SiGe_	2 nm
Germanium mole fraction	x	0.4
Gate-oxide (SiO_2_) thickness	t_ox_	1 nm
Source doping	D_s_	4.5 × 10^19^ cm^−3^
Drain doping	D_d_	3 × 10^19^ cm^−3^
Channel doping	D_ch_	1 × 10^15^ cm^−3^
Channel length	Lg	10 nm
Source/drain/channel radius	r_s_, r_d,_ r_ch_	4 nm

**Figure 3:**
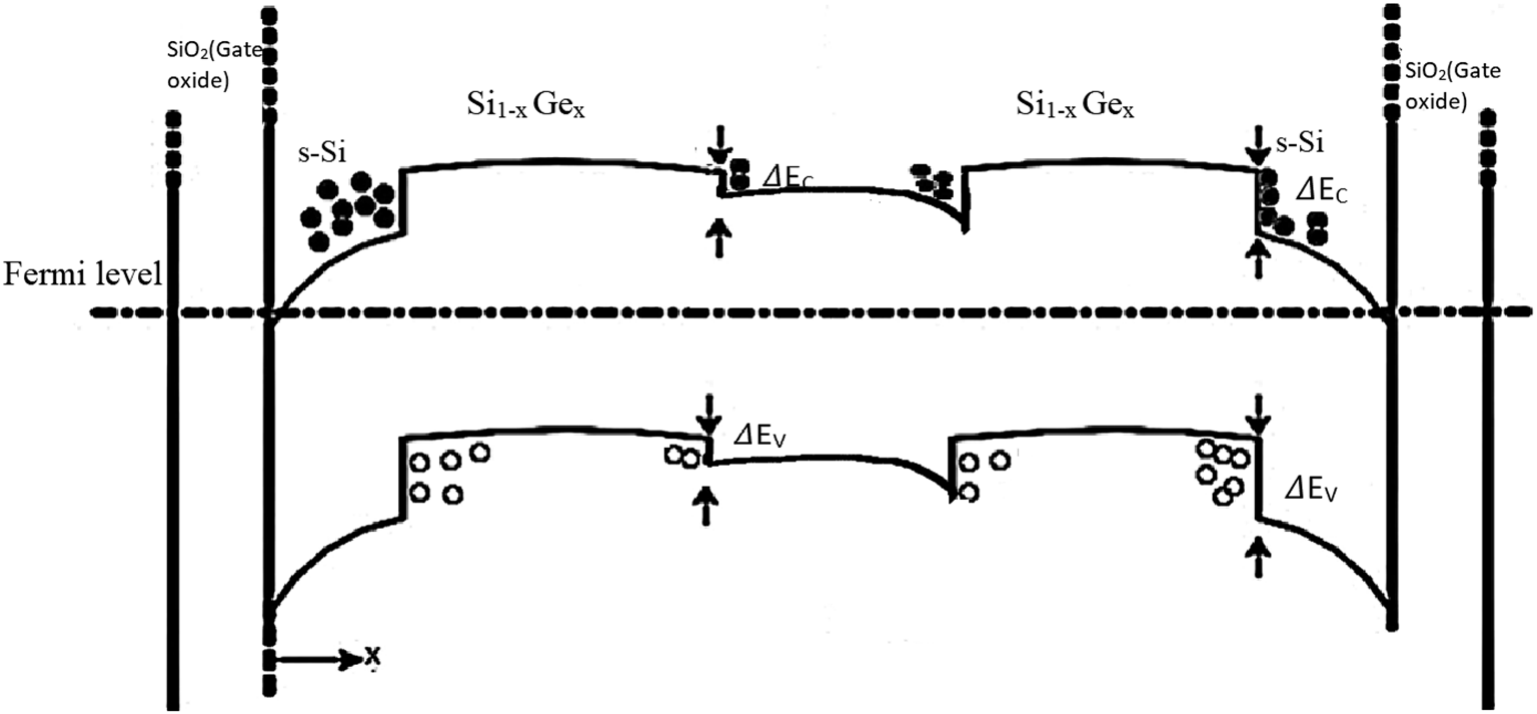
Depiction of carrier confinement and transport mechanism occurring at quantum wells of the tri-layered strained channel CGAA FET**.**

Considering the strain model for the CGAA channel, bandgap narrowing, quantum carrier confinement and transition of carriers via quasi-ballistic transport occurs in the channel system that are simultaneously included for the detailed analysis of the innovative NW CGAA. According to observations, the device has a Type-II hetero-band system that includes the strain effect and carrier confinement effect (see Fig. 3). This results in the creation of a cylindrical Nano-system with a tri-layered channel induces quasi-ballistic transport phenomena via an incredibly thin barrier, illuminating the potential construction of a charge centroid due to the 2D electrostatic potential difference. Schrodinger and Poisson's equations are concurrently solved at the junction of the outer s-Si layer and gate oxide layers, which results in electron confinement at potential barriers^26^. This causes the energy bandgap between the permitted energy level to widen in a relatively thin region, eventually counterbalancing the strain and possibly reducing SCEs with performance improvements. The conduction band divides into twofold and four-fold valleys due to band splitting of the s-Si and s-SiGe layer based on Type-II heterogeneity, resulting in drop in effective mass, thereby increasing the effective charge carrier mobility^27^. Due to the device symmetry, the Poisson’s equation of the strained channel CGAA MOSFET is written as:

5 $$\frac{1}{x}\Delta \left(x.\Delta \varphi \left(x,z\right)\right)+{\Delta }^{2} \varphi \left(x,z\right)={qN}_{A}/ {{\mathcal{\rm P}}}_{s{\text -}Si}, \quad 0\le \mathrm{ x }\le \mathrm{ R}, 0\le \mathrm{ z }\le \mathrm{Lg}$$

where φ(r,z) is the electrostatic potential in the channel, ${\mathcal{\rm P}}$_s-Si_ is strained silicon permittivity. A parabolic approximation of tri-layered channel potential distribution in radial direction is applied for the solution of Eq. (5), and is given by:

6 $$\varphi \left(x,z\right)={A}_{0}\left(z\right)+{A}_{1}\left(z\right)x+{A}_{2}(z){x}^{2}$$

where the coeffiecients A_0_(z), A_1_(z), and A_2_(z) are functions of channel length in 3D, which is quantified considering the boundary conditions for the novel device:

i. Assuming an electrostatic surface potential at the gate oxide and outer-strained silicon interface to be:
  7 $$\varphi \left(x,z\right){|}_{x=R}={\varphi }_{s}\left(z\right)$$
ii. Inner s–Si forms a well at the device centroid, therefore is the virtual substrate in the channel acquiring negligible carrier denisty with zero electric field, hence is given by:
  8 $$\varphi (x,z){|}_{x=0}=0$$
iii. The electric field is continuous at the interface between the gate oxide and outer s-Si and is given as:

  9 $$\Delta (x,z){|}_{x=R}=\frac{{C}_{ox}^{\prime}}{{{\mathcal{\rm P}}}_{s{\text -}Si}}\left[{V}_{GS}-{\left({V}_{FB}\right)}_{s{\text -}Si}-{\varphi }_{s}\left(z\right)\right]$$

On applying the boundary conditions Eqs. (7)–(9) in Eq. (6), the coefficients are determined and therefore adjudged as:

10 $$\mathrm{ \varphi }\left(\mathrm{x},\mathrm{z}\right)={\mathrm{\varphi }}_{\mathrm{s}}\left(\mathrm{z}\right)-\frac{{\mathrm{xC}}_{\mathrm{ox}}^{{{\prime}}}}{2{\mathrm{{\mathcal{\rm P}}}}_{\mathrm{s}-\mathrm{Si}}}\left[{\mathrm{V}}_{\mathrm{GS}}-{\left({\mathrm{V}}_{\mathrm{FB}}\right)}_{\mathrm{s}-\mathrm{Si}}-{\mathrm{\varphi }}_{\mathrm{s}}\left(\mathrm{z}\right)\right]+\dots \frac{{\mathrm{C}}_{\mathrm{ox}}^{{{\prime}}}}{2{\mathrm{R{\mathcal{\rm P}}}}_{\mathrm{s}-\mathrm{Si}}}\left[{\mathrm{V}}_{\mathrm{GS}}-{\left({\mathrm{V}}_{\mathrm{FB}}\right)}_{\mathrm{s}-\mathrm{Si}}-{\mathrm{\varphi }}_{\mathrm{s}}\left(\mathrm{z}\right)\right]{\mathrm{x}}^{2 }$$

In this N-channel novel CGAA FET (Device A), electron is the majority carrier transport factor, and SiGe being a 2 nm thick barrier layer due to Type-II heterogeneity, quantum tunneling is neglected, so the potential is not considered. The electric field distribution is calculated along the x direction as:

11 $$E\left(x,z\right)=-\frac{d\varphi \left(x,z\right)}{dx}=\frac{{C}_{ox}^{\prime} .x}{R{{\mathcal{\rm P}}}_{s{\text -}Si}}{\varphi }_{s}\left(z\right)$$

where gate oxide capacitance, ${C}_{ox}=\frac{{\mathcal{ \rm P}}_{ox}}{{t}_{ox}}$ is further given as ${C}_{ox}^{\prime}=\frac{{{\mathcal{\rm P}}}_{ox}}{R\mathrm{ln}\left(1+{t}_{ox}/ R\right)}$. Surface potential at gate-oxide contact is φ_s_ (z), and ${\mathcal{\rm P}}$_ox_ represents the permitivity. As a result of the strain, (V_FB_)_s-Si_ is produced and is estimated to be:

12 $${\left({V}_{FB}\right)}_{s{\text -}Si}={\left({V}_{FB}\right)}_{si}+\Delta { V}_{FB}$$

where (V_FB_) = Φ_M_ − Φ_si_,

$$\Delta {V}_{FB}=-\frac{(\Delta {E}_{c}{)}_{s{\text -}Si} }{q}+\frac{(\Delta {E}_{g}{)}_{s{\text -}Si}}{q}-{V}_{T }\mathrm{ln}\left(\frac{{N}_{Vsi}}{{N}_{Vs{\text -}Si}}\right),$$

(V_FB_)s–si and (V_FB_)_si_, respectively, are the flat-band voltages of strained silicon and silicon. Φ_M_ stands for metal while Φ_si_ stands for silicon work function. N_vsi_ and N_vs-si_, respectively, are effective density of states of silicon and s–Si in valence band. V_T_ = kT/q is the thermal voltage. On calculating the flat band voltage of the channel, the threshold voltage is further estimated for the novel CGAA device and is given as:

13 $${{V}_{th}}_{s{\text -}Si}=\frac{\left|{Q}_{SD}^{{{\prime}}}(max)\right|}{{C}_{ox}}+{{V}_{FB}}_{s{\text -}Si}+2{{\Phi }_{fn}}_{s{\text -}Si}$$

where ${Q}_{sd}^{\prime}(\mathrm{max})=e{N}_{d}{x}_{dt}$, ${{\Phi }_{fn}}_{s{\text -}Si}={V}_{T}\mathrm{ln}( \frac{{N}_{D ch}}{{n}_{in s{\text -}Si}} )$, ${x}_{dt}$ is the space charge width, N_Dch_ is the donor doping concentration of the tri-layered channel Nano-system, and n_ins-si_ is carrier concentration of inner s-Si. The threshold voltage for the Device A is therefore calculated to be 0.3 V. Considering this the related parametric analysis of the strained tri-layered NW CGAA FET is carried out using Silvaco tool^28^. Based on the actual device realization the Band Gap Narrowing model, the Lombardi CVT model, the SRH model, Auger model and Arora model along with Hansch quantum models are employed to study and investigate the performance analysis that nurtured on the device parameters such as current density effect, electron mobility, electric field, electrostatic potential, and electron velocity. Band Gap narrowing model accounts the band narrowing effects in channel due to the induced strain, while CVT and SRH models incorporate the mobility analysis and parameterization in the novel device. Using concentration, temperature, parallel field, and transverse field dependence, CVT establishes a general-purpose mobility model and thereby energy balance is maintained to develop the nano channel length devices. The drift diffusion model of the Silvaco tool^28^ is also inducted where Poisson's equation and the electron continuity equation are solved self-consistently to investigate the ballistic charge transport through the channel. Derivations based upon the Boltzmann transport theory and current density is approximated by the drift–diffusion model and hence is attractive by not introducing any independent variables. In this ultra-thin strained channel device, Hansch’s quantum model accounts the carrier confinement effects in the inversion layers and presents a quantitative estimation of the probable enrichment in mobility, potential and electron velocity in the device. The ON current and finally the output drive current across the channel are thereafter estimated and a qualitative analysis is overlaid for the novel NW CGAA FET.

## Results and discussion

**Figure 4:**
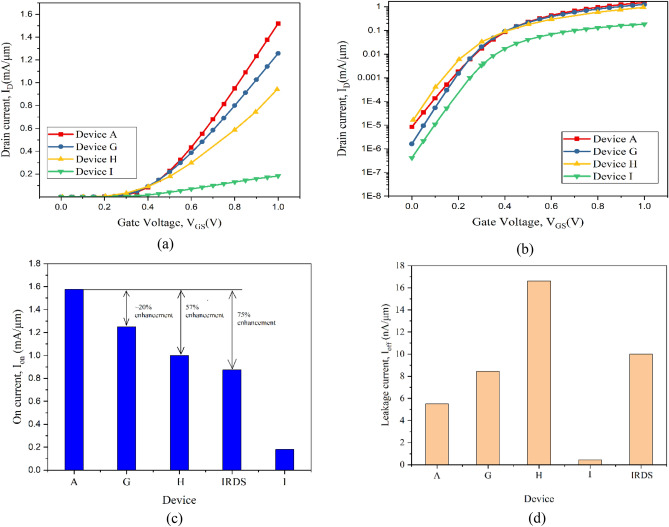
(**a**) I_D_–V_GS_ characteristic at V_DS_ = 1 V validating base Device A with existing Device H and Device I, while healthier performance of Device A is perceived. (**b**) Logarithmic representation of I_D_–V_GS_ characteristic, while healthier performance of Device A is perceived. (**c**) ON current of the devices presented providing ~ 57% enhancement for Device A (10 nm CGAA NW FET) over Device H (conventional 22 nm CGAA FET. (**d**) Leakage current of the Device A (10 nm CGAA NW FET) with existing Device H and Device I, While Device A providing leakage current within the acceptance limit of IRDS 2022.

The Strained Channel NW CGAA FET (Device A) is developed here for the first time with tri-layered nano-channel (s-Si/s-SiGe/s-Si) system having radial thicknesses as described in Table 1 in the form of 1–2–1 nm considering the calculated V_th_ in “Device structure and theory” is nearly equal to acquired threshold voltage 0.29 V. In order to minimize the impacts of short channel effects and maintain a low threshold voltage, the overall channel thickness is fixed at half the length of the gate (10 nm). The base device (Device G) is of 22 nm gate length strained channel CGAA FET, while the silicon channel conventional CGAA FET of 22 nm gate length is Device H^16^ and the tri-layered HOI FinFET of 10 nm gate length is Device I^22^ are the reference devices for further investigation and analysis of the present work. The I_D_–V_GS_ characteristics as acquired in Fig. 4a, designates enriched performance for Device A. The occurrence of quasi-ballistic carrier transport and sustenance received from the confined carriers (witnessed in Fig. 3) of the strain induced ultrathin s-Si wells of the Nano-system gadget substantiates this experiential presentation. This condition of ballistic transport and carrier confinement is attributed to the development and incorporation of Type-II heterostructure system in the nano-channel device, which is the first of its kind and results in a novel phenomenon of succumbing of quantum carriers with the formation of charge centroid leading to superior enactment for Device A. This physical portfolio is not acquirable in longer channel length (Device G) due to nonexistence of ballistic transport, and cannot be measured in Device I as it suffers from current crowding corner effects with reduced control for tri-gate rectangular structure, while it is not applicable in the conventional Device H as strain system does not exist in Si only CGAA FET. The leakage current is anonymously detected from the logarithmic I_D_-V_GS_ characteristics of Fig. 4b and the leakage current plot of Fig. 4d inevitably showcasing lower leakage for Device A. On shortening channel length, the leakage current increases in Device A due to SCEs. Despite the fact that I_off_ for the 10 nm channel length NW device has a greater leakage the data is well within the permitted range of the 3 nm technology node device as accorded by IRDS 2022^19^, hence Device A meets the requirement and is at par with the recent technological challenges.

The ON current (I_on_) analysis is carried out for Devices A, G, H, and I which are assimilated to be 1.57 mA/m, 1.26 mA/m, 1.0 mA/m, and 0.183 mA/m, respectively, and are presented in Fig. 4c. The figure inevitably depicts that Device A surpasses all the other three devices, indicating to be the supreme most device at the particular regime. Device H^16^ being the conventional Si CGAA FET is compared for validation with Device G (strained CGAA FET) and at 22 nm gate length ~ 20% enhancement in ON current is attained attributing to the inculcation of the tri-layered strain effect in the hetero-channel system. The s-Si layers under global strain effect therefore stretches the atomic structure leading to a smooth passage for electrons by negligible scattering effect thereby increasing the mobility. To this effect as the shorter gate length of 10 nm is inducted, the quasi-ballistic transport of carriers is also gripped through the s-Si regions in the novel tri-layered strained channel NW CGAA FET (Device A) thereby, showcased an emphatic enrichment of 57% when compared to Device H^16^ and 75% in contrast to the 3 nm technology node proposal of IRDS 2022^19^, which is undoubtedly an astounding achievement. In Device A along with hetero-strain effect as of Device G, quasi-ballistic carrier transports are hatched by the ultra-thin s-Si well regions of the channel due to quantum carrier confinement in the Nano-system, resulting in negligible scattering effects thereby leading to higher carrier mobility and enhanced I_on_ is perceived. The leakage current (I_off_) of the novel CGAA FET (Device A) is 5.5 nA/µm, which is higher than Tri-gate FinFET^22^ due to its cylindrical structure but, the leakage current is well within the acceptable limits according to the IRDS 2022^19^ at 3 nm technology node as apparent from Fig. 4c. Hence, the novel 10 nm ultra-thin strained channel CGAA FET can be considered to be an improved alternative since it enhances the on-current and gives better I_on_/I_off_ ration as compared to IRDS 2022 at 3 nm technology node and the existing Trigate Fin FET.

**Figure 5:**
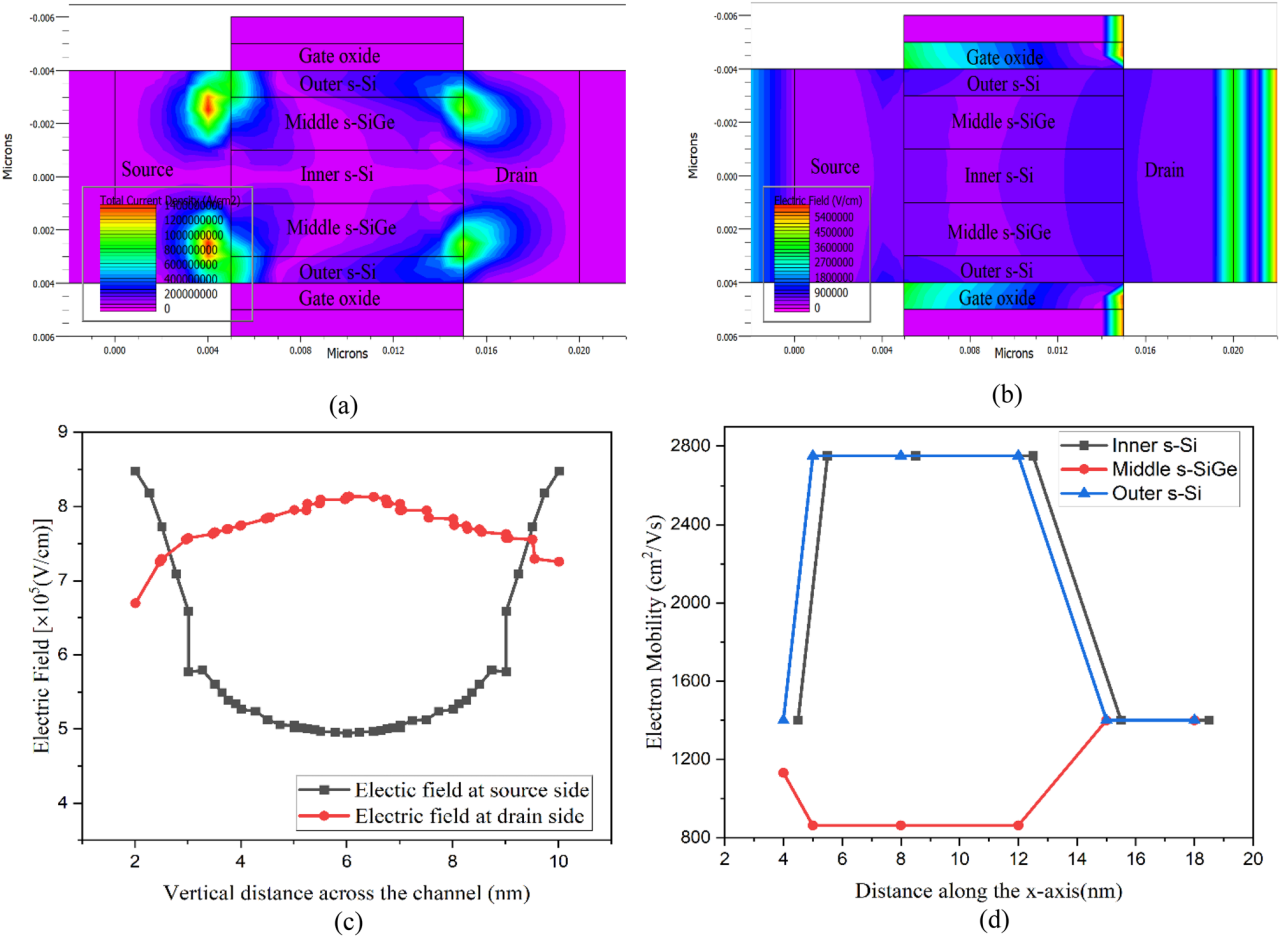
(**a**) Current density contours at the source- and drain-channel interfaces observed in the 10 nm nano-system CGAA FET. (**b**) Electric field contours observed with drain side showing higher E-field. (**c**) Electric field variations for drain side and source side observed vertically across the 10 nm strained channel CGAA NW FET. (**d**) Electron mobility variation across the strained channel CGAA FET for the 10 nm gate length device.

The current density exploration of the tri-layered strained channel Nano-system device leads to the observation as depicted in Fig. 5a. Both the source-channel and drain-channel interfaces of the outer s-Si to the middle silicon germanium layer visualize high current density contours, indicating substantial current density variation across the channel region. On the drain side interface, a relatively sharp rise in current density is vehemently witnessed, due to the Type-II hetero band-alignment of the Nano-system channel, leading to confinement of electronic carriers occurring in the well regions, which contribute to electrostatic centroid charge confinement as in Fig. 3, attributing to the observance of higher current densities at the drain interfaces of the novel strained tri-layered channel NW CGAA FET. This current density rise near the drain end indicates higher electron mobility, which can result in enhanced drain current and in turn the enriched device performances, which is the requisite for developing the NW CGAA FET.

The electric field (EF) contours with variations throughout the vertical channel region are pragmatically detected in Fig. 5b for the ultra-thin channel of the NW CGAA FET. Apparently higher electric field is professed towards the drain end in comparison to the source across the vertical plane of the Nano-system channel as in Fig. 5c, which indicates the greater flow of carriers to the drain attributing to higher source doping. Also, the assessment provides a glance that the EF quite similarly varies in all three regions (outer s-Si, middle S-SiGe and inner s-Si) of the channel, while it is to be noted that the EF is stronger at the inner s-Si layer that decreases towards the middle s-SiGe followed by the outer s-Si layer, which is further exemplified from the EF-curve of Fig. 5c. This variation accounts to the effect of transverse field applied through the gate to channel of the device. The deviation boons an indication that quasi-ballistic transport of carriers is inferior through the inner s-Si layer of the channel and is due to diminished tensile strain effect along with lessened gate control occurring on the inner s-Si layer, which subdues as the virtual substrate in the tri-layered strained channel NW CGAA FET. The electric field at the drain side (red line) is not symmetric due to asymmetric doping of source and drain regions. In this novel device the strain on the tri-layered system causes band bending in the channel area and leads to splitting in fourfold and twofold valleys in the heterostructure, thereby loosens the compactness of Si molecules to become tensile strained Si, hence increasing the electron mobility^3^. Due to the channel length being short (10 nm) for devices A to F developed here, ballistic transport overrules and negligible roughness scattering inducts as is clearly observed from the mobility graphs (Fig. 5d)showing no much variation in the channel for the transport of electrons from source to drain through the strained-Si layer, while slight reduction in carrier mobility is observed in line with the perception of^29,30^ due to the influence of negligible roughness at the strained-Si–SiGe interface. In this novel device the strain on the tri-layered system causes band bending in the channel area, initiating energy band splitting and increasing occupancy of electrons in twofold valleys of the heterostructure, which in turn congregates in enhanced electron mobility, suppressing the intervalley transition of electrons from lower valley to upper valley reducing the phonon scattering in the ultrathin channel. Figure 5d illustrates the variance in electron mobility across the 10 nm Nano-system CGAA FET (Device A). Due to hole confinement, as deceptively seen in Fig. 3, the middle (stained-SiGe) layer is where hole carrier transport predominates, while electron mobility at the source-channel interfaces substantially increases for both outer and inner s–Si layers. The mobility remains constant throughout the length of the channel signifying negligible scattering effect leading to ballistic transport of carriers occurring through the channel region of the NW device, devising boosted output performances. The comparison of electrical characteristics of the 22 nm and 10 nm channel length devices G (this work) and A (this work) is compared with the CGAA established by Karabelai et al.^16^, the 10 nm TG HOI FinFET developed by Nanda et al.^22^ and presented in Table 4. The ON current of A is determined to be far superior to all other devices along with minimalistic variations in leakage currents and other SCEs, thereby installing A as the device of the future.

**Table 4:** Comparison of NW channel FET electrical characteristics.

Electrical Parameters	Karabelai et al.^16^	Nanda et al.^22^	IRDS 2022^19^	This work [A]	This work [G]
V_th_ (V)	0.2	0.25	0.15	0.29	0.30
I_on_ (mA/μm)	1	0.183	0.874	1.57	1.26
I_off_ (nA/μm)	16.6	0.102	10	5.5	8.45
I_on_/I_off_ ratio	60,241	179,444	87,400	286,156	780,096
SS (mV/dec)	76	82.06	82	79	65.25

**Figure 6:**
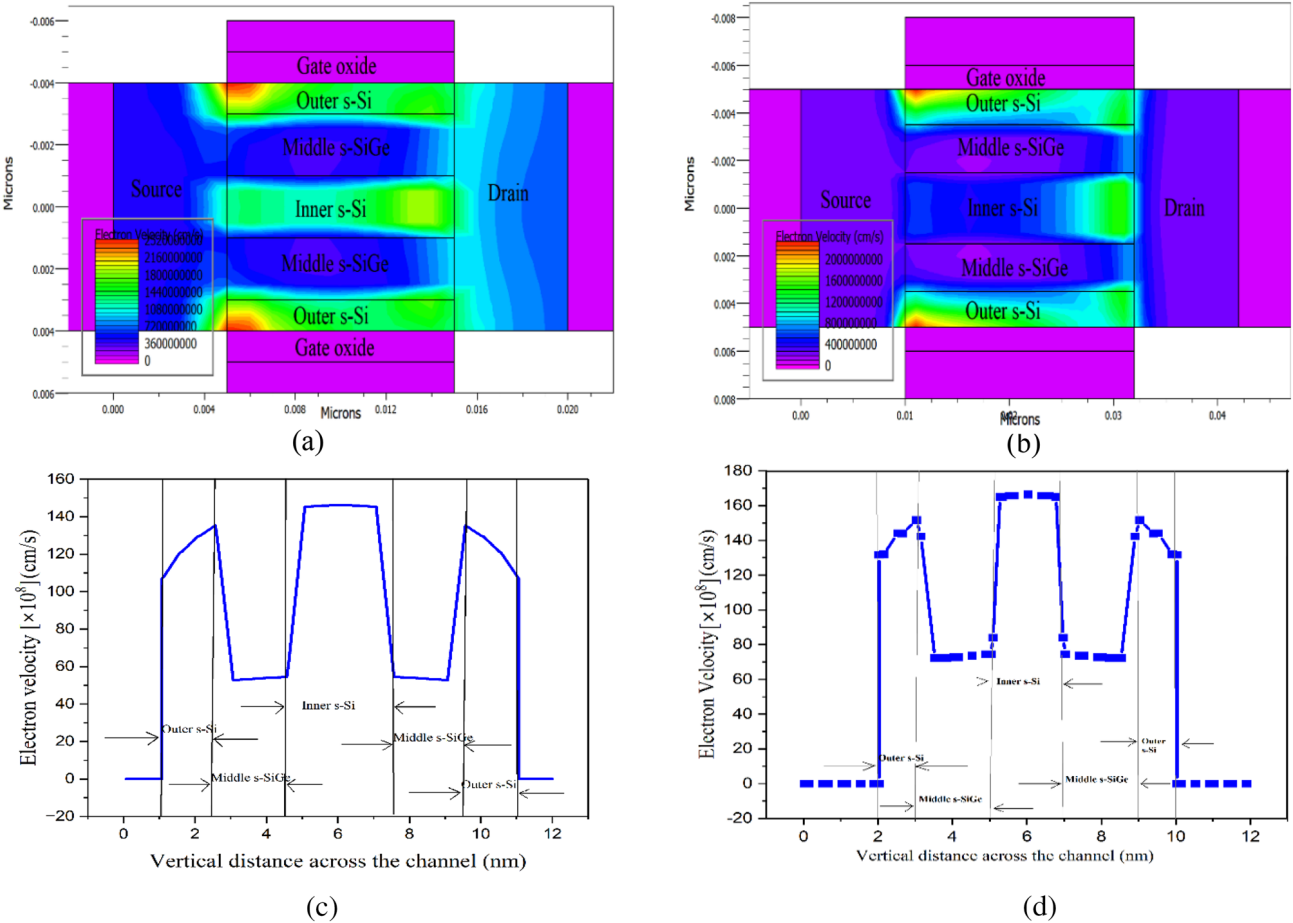
(**a**) Electron velocity contours observed across the 10 nm strained channel CGAA FET (DeviceA). (**b**) 22 nm CGAA FET (Device G) electron velocity contours in s-Si layers revealed vertically across the NW channel. (**c**) Electron velocity variation observed across the 10 nm strained channel CGAA FET (Device A). (**d**) Electron velocity variation observed across the 22 nm CGAA FET (Device G), higher electron velocity in s-Si layers revealed vertically across the NW channel.

**Figure 7:**
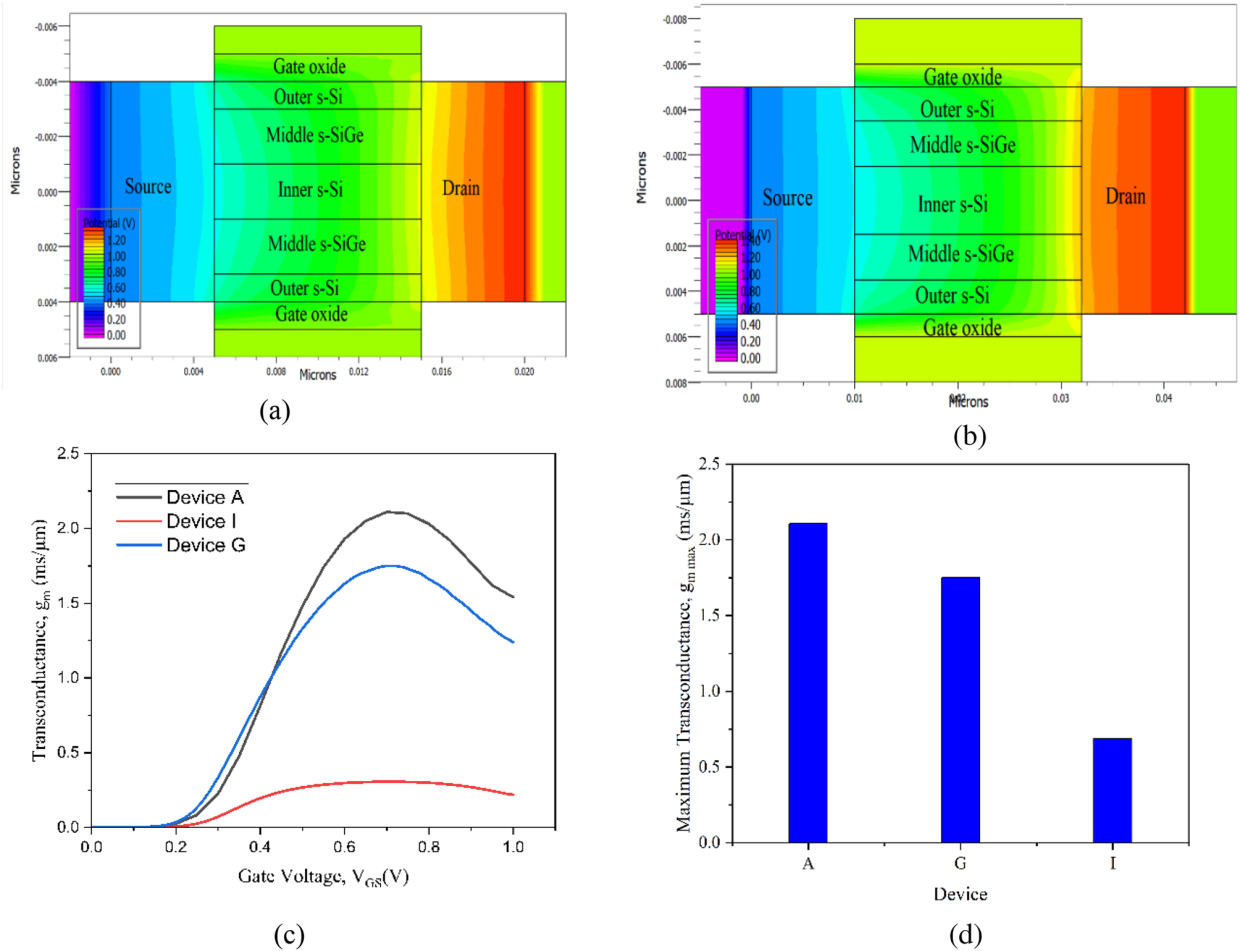
(**a**) Electrostatic potential contours observed across the 10 nm strained channel CGAA FET. (**b**) Electrostatic potential contours observed across the 22 nm strained channel CGAA FET. (**c**) Transconductance (g_m_) of Device A and G at 0.5 V drain voltage with existing device I. (**d**) Maximum transconductance comparison providing healthier performance for Device A over Device I^20^.

Figure 6a divulges the electron velocity across the NW channel of the 10 nm CGAA FET (Device A) while Fig. 6b reveals the electron velocity across the NW channel of the 22 nm CGAA FET (Device G). The electron velocity is observed to slightly increase from the source to the drain end interface of the channel as also detected for electric field from Fig. 5b sufficing higher source doping. Both the outer and inner s–Si layers of Fig. 6a, b equally demonstrate significantly huge velocity of electrons passing through the Nano-system channel of the device in contrast to the middle s-SiGe barrier layer, which is further exaggerated by the cut line analysis graphically witnessed in Fig. 6c, d for Device G and Device A respectively. Attributing to the tensile strain effect of the Si layers, SiGe is more prone to hole carrier transport, thus the electron mobility in-turn the electron velocity is havoc in the s-Si layers in contrast to the s-SiGe (middle) layer. This tops with the Type-II heterogeneity formed in the Nano-system of the NW channel of the device leading to band bending and splitting occurrences essentially improving the electron mobility through ballistic transport, which is well supported and detected by the electric field and electron mobility of Fig. 5c, d, respectively. It is to be noted that for both the devices the electron velocity is quite low in the inner s-Si layer in comparison to the outer s-Si layer, which vehemently signifies that the transport of carriers is higher in the outer layers attributing to the strain effect and inner s-Si more prone to act as the virtual substrate of the NW CGAA FET. This condition of electron velocity and its effect induces probable drive current enrichment in the novel CGAA NW device. On inspecting, electrostatic potential contour waves are observed in Fig. 7a, b across the channel of the 10 nm CGAA NW FET and 22 nm CGAA NW device respectively, which adheres to electric field and) electron velocity investigation that increases gradually from the source-end to the drain-end interface in the Nano-system channel region and is based on the flow of carriers from the source to drain. This situation along with carrier confinement in the Type-II hetero band-alignment of the channel contributes to the foundation of 2D electrostatic charge centroid in the channel, causing enhancement in drain current of the nanodevice.

The transconductance (g_m_) analysis of the CGAA NW device is presented in Fig. 7c, which is the key test for validating the nFET performance and is calculated by:

14 $${g}_{m}=\frac{\partial {I}_{D}}{\partial {V}_{g}}$$

The plot for the maximum transconductance of devices is presented in Fig. 7d, where NW strained channel CGAA showcases maximum g_m_ of 2.11 mS/m for the 10 nm gate length device. This strained channel system in the CGAA FET is introduced with the tri-layered strain combination leading to the formation of wells with Type-II hetero-band structure at nano regime. This novel Nano-system initiates improvement in carrier mobility and electric field of the device as is observed from the analysis due to congealment of quasi-ballistic transport and carrier confinement in the NW channel region. Hence, the surface-parallel carrier transport properties enhance in upgraded transconductance for the device. As a result, the transconductance increases, and a plausible increase in drain current is predicted from Fig. 7c for the cylindrical GAA FET with strained channel Nano-system.

**Figure 8:**
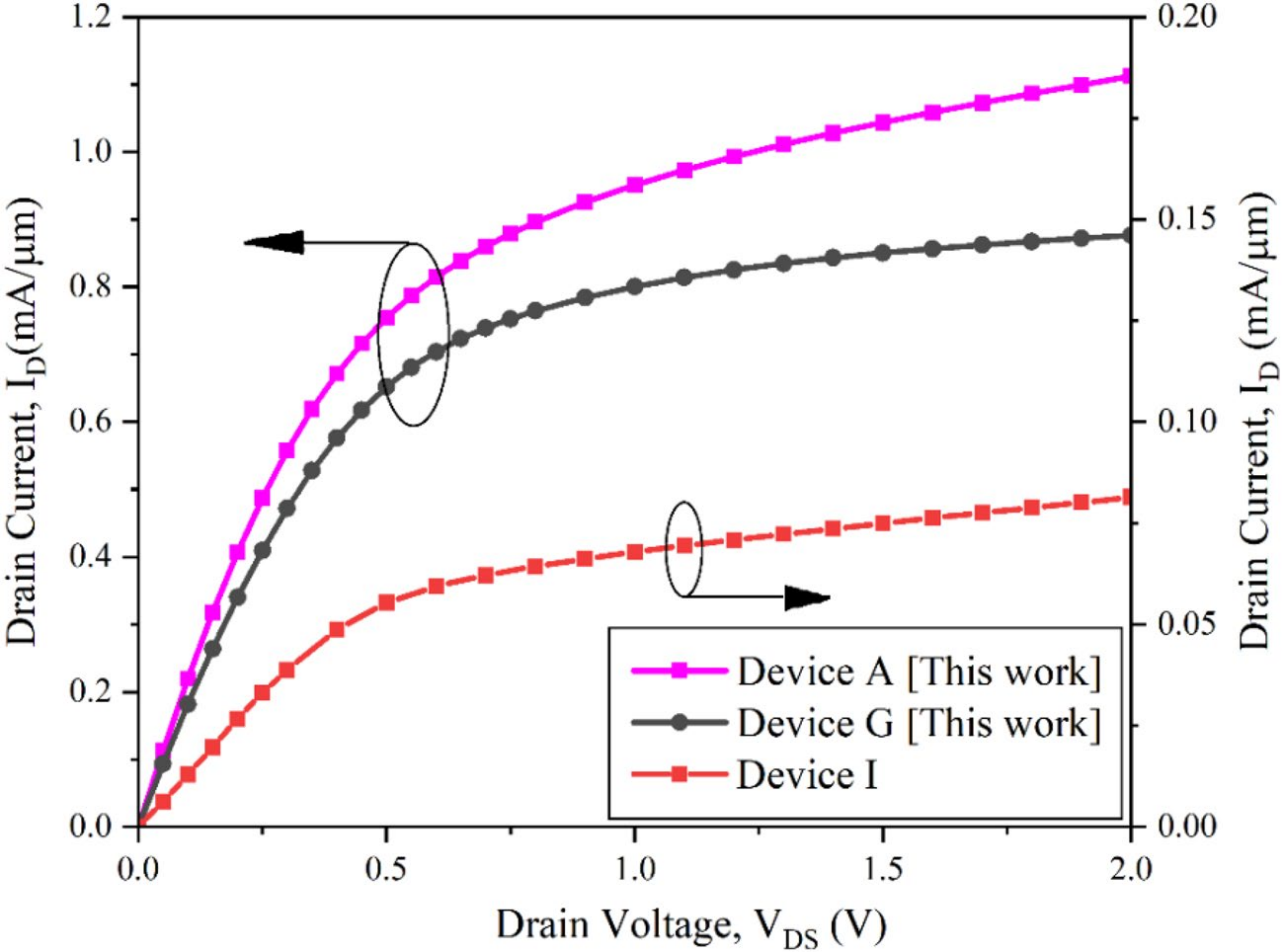
I_D_–V_DS_ characteristics at V_GS_ = 0.8 V for Device A, Device B and Device D at V_GS_ = 0.6 V compared for enhancement performance.

In continuation to the investigation and analysis, the I_D_–V_DS_ characteristics is acquired and contrived in Fig. 8 presenting the output performance of the device. Device A contributes to ~ 28% enhancement in drain current as compared to Device G, thus by shortening the gate length to 10 nm the mobility of carriers increases vigorously due to the Type-II band alignment and strain effect in the cylindrical channel region. This leads to quasi-ballistic transport effects based on 2D electrostatic charge potential happening in the shortened NW device, which is further supported by the carrier confinement as detected in Fig. 3 in consonant to the novel phenomenon of succumbing of quantum charge carriers in the channel. Considering these effects, the 10 nm gate length NW CGAA subdues ballistic transit of charge carriers, resulting in augmented drain current and enriched device performances. Bearing this aspect, a comparison analysis of Device A and Device I is inspected and a contribution of sixfold improvement on drain current at 0.6 V gate voltage is perceived for Device A. Hence, this novel device stands as the state-of-the-art device, ready to meet the requirement for enriched drain current performances with controlled leakages via all-round double-strain effect on inducing Type-II hetero-band alignment in the NW system leading to quasi-ballistic transport and supplemented mobility in the device.

Device-A therefore outshines the performance with the initiation of the tri-layered strained technology forming the novel NW CGAA architecture. Ballistic carrier transit occurs due to quantum carrier confinement and quasi ballistic transport in the strained channel layers, which results in enhancement of carrier mobility and counter threshold voltage roll-off leading to enriched electric field, electron velocity and transconductance with reduction in the leakage current acquired by gate-all-around architecture attributing to negligible current crowding and scattering effects, thereby augmented on-current with lessened SCEs are observed elevating the overall device performance.

## Conclusion

A tri-layered strained channel Cylindrical Gate-all-around Nanowire (CGAA NW) FET at 10 nm gate length is developed and analyzed here for the first time with calculated V_th_ = 0.29 V for the device. The ultra-thin strained channel of the novel device (Device A) embraces Type-II hetero-band alignment forming the Nano-system with s-Si wells through the length of the cylindrical channel, which oversees enhancement of carrier mobility by quasi-ballistic transport and quantum carrier confinement, while reduction of the leakage current is achieved by the GAA architecture. Device A provides ~ 20% enhancement in ON current with respect to Device G (22 nm gate length device) and 57% improvement when compared to the 22 nm silicon-only channel CGAA FET (Device H) and 75% enhancement in contrast to the 3 nm technology node IRDS 2022 proposal. Device A also provides 3.7% decrement in subthreshold swing and 227% enhancement in I_on_/I_off_ ratio when compared to IRDS 2022 for the 3 nm technology node, which is quite significant and is exceedingly augmented in contrast to Si-only Device G. This is attributed to the induction of Type-II hetero-band with strain effect along with charge centroid creation instituted electric field and electrostatic potential in the s–Si layers of novel device. Thereby, enhanced on-current, electron velocity and transconductance are observed for the novel device while reduction in the SCEs and enriched overall device performance is perceived. With the addition of strained tri-layer Nano-system in the channel region (Device A) with shortened gate length (10 nm), a remarkable drain current enhancement of ~ 28% is attained for the output performance in contrast to Device G due to the occurrence of carrier confinement and quasi-ballistic transport of carrier in the channel region. This novel CGAA NW FET with strained Nano-system channel thereby confirmed its competence to meet the sceintific requirements on achieving faster operating speed with minimal SCEs and is thus the most apposite device for the future technological era.

## Data Availability

The datasets used and analyzed during the current study are available from the corresponding author on reasonable request.
